# iEnhancer-EBLSTM: Identifying Enhancers and Strengths by Ensembles of Bidirectional Long Short-Term Memory

**DOI:** 10.3389/fgene.2021.665498

**Published:** 2021-03-23

**Authors:** Kun Niu, Ximei Luo, Shumei Zhang, Zhixia Teng, Tianjiao Zhang, Yuming Zhao

**Affiliations:** ^1^College of Information and Computer Engineering, Northeast Forestry University, Harbin, China; ^2^School of Computer Science and Technology, Harbin Institute of Technology, Harbin, China

**Keywords:** enhancer, identification, classification, recurrent neural network, long short-term memory

## Abstract

Enhancers are regulatory DNA sequences that could be bound by specific proteins named transcription factors (TFs). The interactions between enhancers and TFs regulate specific genes by increasing the target gene expression. Therefore, enhancer identification and classification have been a critical issue in the enhancer field. Unfortunately, so far there has been a lack of suitable methods to identify enhancers. Previous research has mainly focused on the features of the enhancer’s function and interactions, which ignores the sequence information. As we know, the recurrent neural network (RNN) and long short-term memory (LSTM) models are currently the most common methods for processing time series data. LSTM is more suitable than RNN to address the DNA sequence. In this paper, we take the advantages of LSTM to build a method named iEnhancer-EBLSTM to identify enhancers. iEnhancer-ensembles of bidirectional LSTM (EBLSTM) consists of two steps. In the first step, we extract subsequences by sliding a 3-mer window along the DNA sequence as features. Second, EBLSTM model is used to identify enhancers from the candidate input sequences. We use the dataset from the study of Quang H et al. as the benchmarks. The experimental results from the datasets demonstrate the efficiency of our proposed model.

## Introduction

Enhancers, as cis-acting DNA sequences, are small pieces of DNA that are surrounded by specific proteins that often boost the expression of specific genes, and the specific proteins are always transcription factors (TFs) (Sen and Baltimore, 1986; Krivega and Dean, 2012; Pennacchio et al., 2013; Liu B. et al., 2016, 2018; Nguyen et al., 2019). In fact, enhancers play a highly important role *in vivo*. As we know, enhancers can increase the gene expression by interacting with TFs. By activating the transcription of genes, one way that enhancers influence target gene transcription is by bringing enhancers close to target genes by forming chromatin loops, and the other way is through self-transcription. Either way will bring about increasing of gene expression (Krivega and Dean, 2012). Moreover, it is well known that enhancers can influence human health and many human diseases. Recently, researchers have shown that under evolutionary constraints, approximately 85% of human DNA corresponds to non-protein-coding sequences with a significant portion constituting cis-regulatory elements. It is therefore not surprising that genetic variations within these regulatory sequences may lead to phenotypic variations and serve as the etiological basis of human disease (Shen and Zou, 2020). This indicates that enhancers might contribute to evolution.

As the amount of histone modifications and other biological data available on public databases and the development of bioinformatics, gene expression and gene control have become increasingly well known (Kleinjan and Lettice, 2008; Liu G. et al., 2016, 2018; Liu et al., 2017; Wang et al., 2020), and study about enhancers is a hot spot currently, especially how to identify enhancers and their strength (Zou et al., 2016; Zacher et al., 2017; Zhang T. et al., 2020). However, there remain many challenges to identify enhancers. For example, enhancers locate in the non-coding regions that occupy 98% of the human genome. This feature leads to a large search space and increases the difficulty. It is also a formidable challenge that enhancers are located 20 kb away from the target genes, or even in another chromosome, unlike promoters are located somewhere around the transcription start sites of genes. These features make identifying the enhancers more difficult (Pennacchio et al., 2013). As a result, in recent years, a large number of researchers have turned their attention to this topic. In 2017, Zacher et al. proposed a hidden Markov model named Genomic State ANotation (GenoSTAN), which is a new unsupervised genome segmentation algorithm that overcomes many limitations, such as unrealistic data distribution assumptions. Although the experience has shown that chromatin state annotation is more effective in predicting enhancers than the transcription-based definition, sensitivity (SN) remains poor (Wang et al., 2020). There are also other algorithms that can be used for enhancer identification and classification. Liu et al. built a predictor that has two layers named “IEnhancer-2L,” which is the first predictor that can identify enhancers with the strength information. The authors used pseudo k-tuple nucleotide composition (PseKNC) to encode the DNA sequences and then made full use of support vector machine (SVM) to build a classifier (Liu B. et al., 2016). In 2018, a new predictor called “iEnhancer-EL” was proposed by Bin Liu et al. iEnhancer-EL is formed through k-mer, subsequence profile, or PseKNC and SVM. Then it obtains the key classifiers and final predictor for layers 1 and 2 (Liu B. et al., 2018; Nguyen et al., 2019). This bioinformatics tool is equivalent to an advanced version of iEnhancer-2L and therefore has better performance than Enhancer-2L. Last year, Quang H. et al. proposed a new model called iEnhancer-ECNN that uses both one-hot encoding and k-mer to encode the sequence and ensembles of convolutional neural networks as the predictor. In our view, it has great improvements in many metrics.

In this study, we build a prediction network named iEnhancer-ensembles of bidirectional long short-term memory (EBLSTM) to identify enhancers and predict their strengths at the same time. We use 3-mer to encode the input DNA sequences. Then we predict enhancers by EBLSTM. Although we only use DNA sequence information, the experimental results prove the effectiveness of our method.

## Materials and Methods

### Benchmark Dataset

**FIGURE 1:**
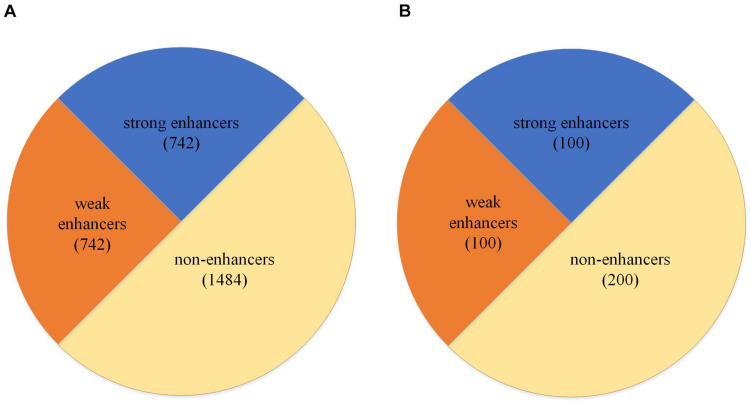
Dataset partition. **(A)** The partition of the development set. **(B)** The partition of the independent test set.

The dataset used in our study is collected from previous studies by Liu B. et al. (2016), Liu B. et al. (2018), and Nguyen et al. (2019) and consists of the chromatin states of nine cell lines, including H1ES, K562, GM12878, HepG2, HUVEC, HSMM, NHLF, NHEK, and HMEC (Liu B. et al., 2016). The dataset is divided into two parts; one part is used to train the model. We called this dataset as the development set. The other part is an independent test dataset. As shown in Figure 1A, the development set consists of 1484 enhancer samples and 1484 negative samples and it is also the layer 1 dataset for enhancer identification. Moreover, 1484 enhancer samples can be divided into 742 strong enhancer samples and 742 weak enhancer samples, and it is the layer 2 dataset for enhancer classification. As shown in Figure 1B, the independent test set contains 200 enhancer samples (100 strong and 100 weak) and 200 negatives. At the same time, the dataset can be presented as follows:

(1) $$D\,a\,t\,a\,s\,e\,t=D\,a\,t\,a\,s\,e\,t_{+}\cup D\,a\,t\,a\,s\,e\,t_{-}$$

(2) $$D\,a\,t\,a\,s\,e\,t_{+}=D\,a\,t\,a\,s\,e\,t_{s\,t\,r\,o\,n\,g}\cup D\,a\,t\,a\,s\,e\,t_{w\,e\,a\,k}$$

where the *Dataset* is all the data that we used, *Dataset*_+_ means the positive dataset, which is the enhancers in our study, and *Dataset*_−_ means the negative dataset, which is the non-enhancer dataset in our study. Therefore, these two formulas mean the *Dataset* consists of *Dataset*_+_ and *Dataset*_−_, and *Dataset*_+_ consists of *Dataset*_*strong*_ and *Dataset*_*weak*_.

**FIGURE 2:**
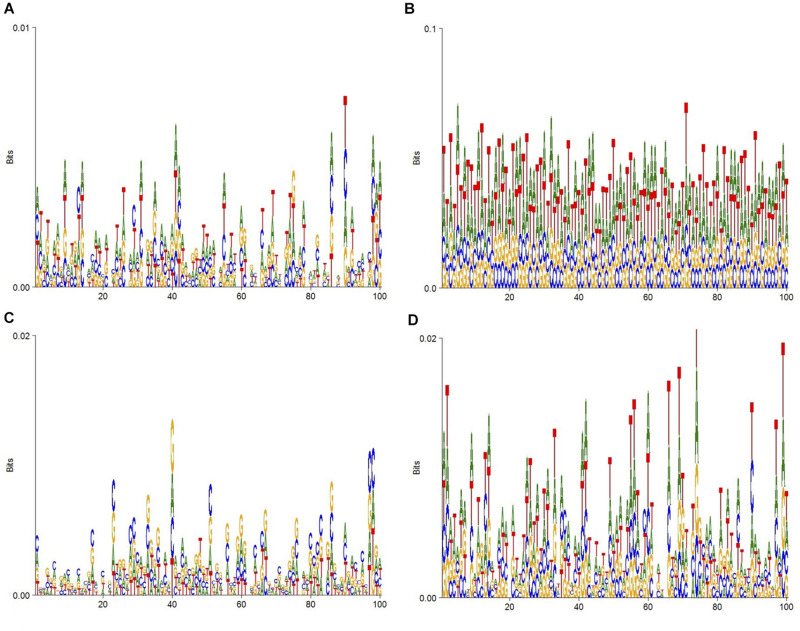
DNA sequence logo. **(A)** The DNA logo of enhancers. **(B)** The DNA logo of non-enhancers. **(C)** The DNA logo of strong enhancers. **(D)** The DNA logo of weak enhancers.

To display the datasets of this experiment more intuitively, DNA consensus sequences of enhancers (Figure 2A), non-enhancers (Figure 2B), strong enhancers (Figure 2C), and weak enhancers (Figure 2D) are calculated. As Figure 2 shows, the specific distributions of A, T, C, and G on these four datasets are different. This means that differences in DNA sequence can be used to distinguish these four types of sequences.

Every enhancer sample has the same length of 200 bp. In the process of building the model, the development set will be divided into five parts, no matter whether in layer 1 or in layer 2, and each part will be the validation in turn and other four parts will be the training set.

### Sequence Encoding Scheme

**FIGURE 3:**
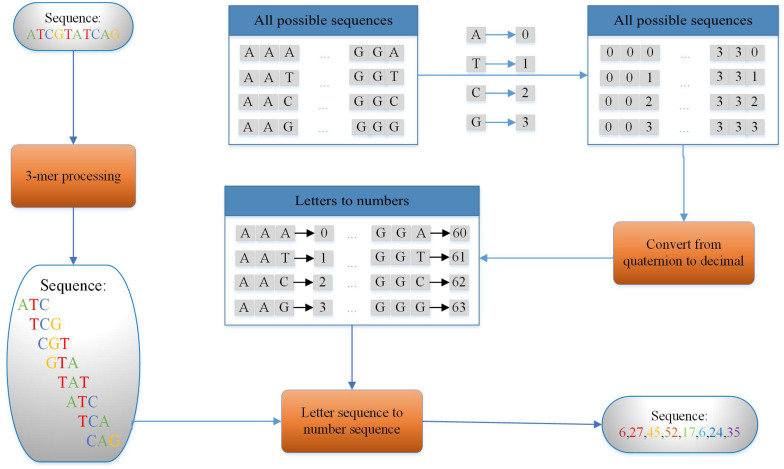
Coding flow of 3-mer (taking DNA sequence with the length of 10 bp as an example).

In this study, we use the principle of k-mer (Liu et al., 2019; Zou et al., 2019; Yang et al., 2020; Zhang Z. Y. et al., 2020), which means dividing the nucleic acid sequence into many shorter subsequences with length of k to encode the 200-bp enhancer sequence. As we know, enhancers are a type of DNA sequence and are composed of two kinds of purines (including adenine and guanine) and two kinds of pyrimidines (including cytosine and thymine). Thus, we can encode the obtained sequence of a length of 200 using k-mer (k = 3) as a sequence with a length of 198 by the encoding method shown in Figure 3. For example, the DNA sequence D is shown as follows:

(3) D={A⁢T⁢C⁢G⁢T⁢A⁢T⁢C⁢A⁢G}

3-Mers are extracted by sliding a 3-mer window along the original DNA sequence with one step as features. The example sequence could be cut into eight such short sequences in S1.

(4) S⁢1={A⁢T⁢C,T⁢C⁢G,C⁢G⁢T,G⁢T⁢A,T⁢A⁢T,A⁢T⁢C,T⁢C⁢A,C⁢A⁢G}

Then, eight numbers are used to represent eight short sequences with a strategy that makes each different 3-bp subsequence corresponds to a different number as shown in Figure 3. The DNA sequence can be transformed as a number sequence as follows:

(5) S⁢2={6,27,45,52,17,6,24,35}

Finally, a number sequence of length 8 can be extracted from a 10-bp DNA sequence. Thus, a sequence of 200 bp in the experiment is encoded in this way and a sequence of 198 digits is produced. Using the sequence ATC in S1 as an example, ATC is regarded as a quaternary three-digit number, A as 0, T as 1, C as 2, and G as 3. Then convert the number in base 3 to base 10. So 64 different 3-mers can be represented by 0–63.

### BLSTM Architecture

**FIGURE 4:**
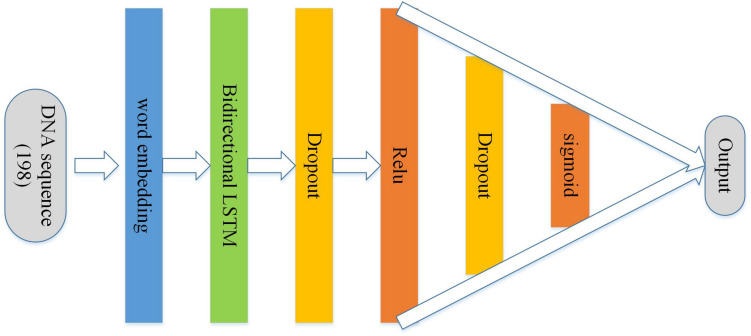
Architecture of the BLSTM model.

As Figure 4 shows, a sequence of numbers with the sequence encoding scheme with the length 198 followed by the body of the structure is used as input to BLSTM. It is mainly composed of an embedding layer, a bidirectional LSTM, a dropout layer, the rectified linear unit (relu), a dropout layer, and sigmoid activation functions. In the architecture, the main purpose of embedding term training is to incorporate into the model to form an end-to-end structure, and the vector trained by the embedding layer can better adapt to the corresponding tasks (Kleinjan and Lettice, 2008; Liu G. et al., 2016, 2018; Liu et al., 2017; Zhang T. et al., 2020). The recurrent neural network (RNN) is a network of nerves that processes sequential data. Compared with the ordinary neural network, it can process the sequence variation data (Zou et al., 2016; Zacher et al., 2017). Long short-term memory (LSTM) is a special RNN, and it is mainly used to solve the problem of gradient explosion and disappearance. In short, LSTM performs better than normal RNN if the sequence is long (Liu et al., 2019; Zou et al., 2019; Yang et al., 2020; Zhang Z. Y. et al., 2020). Bidirectional LSTM is equivalent to the LSTM upgraded version, which means that time sequence data are used to input history and future data simultaneously. In contrast to time sequence, two cyclic neural networks are connected to the same output, and the output layer can obtain historical and future information at the same time (Bian et al., 2014; Goldberg and Levy, 2014; Juntao and Zou, unpublished; Tang et al., 2014). The function of dropout layer is preventing model overfitting. In addition, after relu and sigmoid layers (Gers et al., 1999; Graves and Schmidhuber, 2005; Sundermeyer et al., 2012; Zaremba et al., 2014; Huang et al., 2015; Xingjian et al., 2015; Li and Liu, 2020; Sherstinsky, 2020), a probability of whether the sequence is an enhancer or not can be calculated.

### Ensemble Model

**FIGURE 5:**
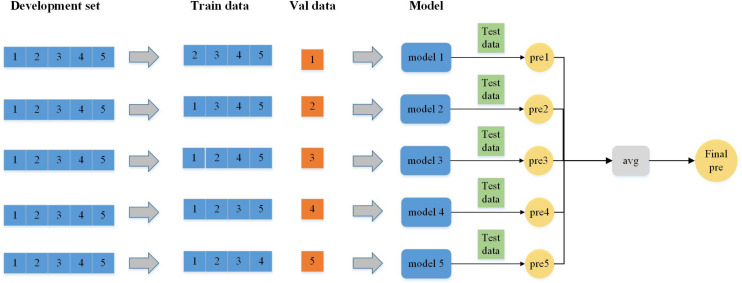
Workflow of the ensemble model (ensemble method is the averaging method).

There are two algorithms in ensemble learning: boosting and bagging (Li et al., 2020; Lv Z. B. et al., 2020; Sultana et al., 2020; Zhu et al., 2020). In our experiment, the data from each experiment are relatively independent and the bagging algorithm is more suitable. First, the basis learner models are trained independently by using subsamples. Finally, the strong learner model is made by different ensemble methods. The testing result shows that bagging is better than boosting. The entire workflow of bagging is in perfect agreement with our experimental procedure. After that, through several experiments, compared with the voting and median methods, the average method (Figure 5) can improve most of the metrics in our experiment in the process of selecting the ensemble method.

In our experiment, the dataset is divided into five parts according to fivefold cross-validation and each part is used as the validation set (Cheng et al., 2019; Dao et al., 2020a; Tang et al., 2020; Zhang D. et al., 2020; Zhao et al., 2020), respectively, and the remaining four parts are used as the training set for the experiment. Five different sets of parameters and models are obtained in these five experiments, and then five sets of models are used to test and obtain the prediction results. The final prediction probability value of the five prediction results is obtained by the average method, and then the prediction results is obtained by comparing with the threshold value of 0.5.

### Measurement

To get the performance of the model, some evaluation metrics are used, such as accuracy (ACC), SN, specificity (SP), Matthews’s correlation coefficient (MCC), and area under the ROC curve (AUC) (Jiang et al., 2013; Cheng, 2019; Liang et al., 2019; Dao et al., 2020b; Lv H. et al., 2020; Shao and Liu, 2020; Shao et al., 2020; Su et al., 2020; Lv et al., 2021; Zhang et al., 2021). In the formulas of these metrics, TP, TN, FP, and FN mean true positive, true negative, false positive, and false negative, respectively. As we know, ACC is a description of systematic errors, a measure of statistical bias, and it always evaluates a model objectively when the dataset is balanced. SN and SP can support the model more accurately when the data are not balanced. The ROC curve is based on a confounding factors matrix, and the abscissa and the ordinate of the ROC curve are the false positive rate (FPR) and true positive rate (TPR), respectively, and AUC is the area under the curve. When comparing the different classification models, the ROC curve of each model can be drawn to obtain the value of the AUC, which can be used as an important indicator to evaluate the quality of a model (Gers et al., 1999; Graves and Schmidhuber, 2005; Sundermeyer et al., 2012; Wei et al., 2014, 2017a,b, 2019; Zaremba et al., 2014; Jin et al., 2019; Su et al., 2019; Ao et al.,2020a,b; Li and Liu, 2020; Sherstinsky, 2020; Yu et al.,2020a,b, c). The higher the AUC value is, the better the model is. The MCC is used as a measure of the quality of binary classifications and it is always used in the field of bioinformatics and machine learning. The reason why it is seen as a balanced measure is that MCC can take into account TP, TN, FP, and FN and we can get more ACC results by this way. MCC is a value between +1 and −1. +1 means a perfect prediction, 0 represents that the method does not work, and −1 indicates that the prediction was the exact opposite. These evaluation metrics are calculated from the count of TP, TN, FP, and FN.

(6) $$A\,C\,C=\frac{T\,N+T\,P}{T\,P+F\,N+T\,N+F\,P}$$

(7) $$S\,N=\frac{T\,P}{T\,P+F\,N}$$

(8) $$S\,P=\frac{T\,N}{T\,N+F\,P}$$

(9) $$M\,C\,C=\frac{T\,P\times T\,N-F\,P\times F\,N}{\sqrt{\left(T\,P+F\,P\right)\,\left(T\,P+F\,N\right)\,\left(T\,N+F\,P\right)\,\left(T\,N+F\,N\right)}}$$

## Results

### Two-Layer Classification Framework

To finish the work in an orderly way, a two-layer classification framework is proposed, which is composed in two steps: identifying enhancer and classifying strong enhancer from weak enhancers. In fact, layers 1 and 2 have the same encoding scheme and network structure. The only difference between the two layers is the input dataset. In layer 1, all data are used as the training set, enhancer set, and non-enhancer set, as part of all data and considered the positive set and negative set, respectively. In layer 2, only the enhancers are used in the experiment. The strong enhancer and weak enhancer are used, respectively, as the positive set and negative set.

#### Layer 1: Enhancer Identification

As we know, enhancer identification is extremely important in the field of enhancers. Now it is a hot topic in bioinformatics. In this study, the process of identification can be regarded as preparation for next step. To illustrate it, before judging whether a DNA sequence is a strong enhancer or a weak enhancer, the first thing is to judge if the sequence is an enhancer or not. If it is an enhancer, then the model predicts if it is strong or weak. Through this process, it becomes easier to understand its characteristics. Compared with layer 2 (enhancer classification), layer 1 will have higher ACC. For the reason, there are more differences between enhancer and non-enhancer than strong enhancer and weak enhancer. The more the difference, the easier it is to distinguish. In the process of the experiment, all of the datasets (enhancer + non-enhancer) are divided into five parts. Data division strategy is shown in Table 1.

**TABLE 1:** The specific division of the dataset into five parts for identifying enhancers and non-enhancers.

Part	Enhancers	Non-enhancers
1	296	296
2	296	296
3	296	296
4	296	296
5	300	300
Total	1484	1484

#### Layer 2: Enhancer Classification

The differences between strong enhancers and weak enhancers are small. Hence, for layer 2, enhancer classification is more difficult than layer 1. Enhancer’s biological function and distinguishing the enhancer’s strength are an important component in understanding its physical and chemical properties. For layer 2, more effort is paid in to study it. In this layer, the enhancer dataset (strong + weak) is split into five parts as layer 1, but the amount of enhancer data is smaller (Table 2). Compared with layer 1, the layer 2 data are characterized by smaller differences and smaller quantities.

**TABLE 2:** The specific division of the dataset into five parts for classifying strong enhancers and weak enhancers.

Part	Strong	Weak
1	148	148
2	148	148
3	148	148
4	148	148
5	150	150
Total	742	742

### Comparison of Different Encoding Schemes

In the second part of our study, we compared the encoding methods that we introduced the sequence and encoding scheme. The encoding method adopted in this article is to encode the letters in the sequence into the numbers by 3-mer. Meanwhile, several other coding methods have also been tested, such as 2-mer, one-hot, and encoding by correspondence between letters and numbers.

k-Mer is obtained by sliding on the DNA sequence with a step size of 1 bp. In our experiment, take 3-mer (k = 3) as an example. When k is 3, 198 3-mers can be extracted from DNA sequence of length 200. Each 3-mer consists of the three letters taken as a whole, so it is possible to encode the original letter sequence into a sequence of numbers of length 198 based on the encoding method shown in Figure 3. In addition, the purpose of k-mer is to enhance the relationship between adjacent letters so that the model can learn better. The same is true for 2-mer, except that we end up with a sequence of 199 digits. Another method is to encode the letters directly in the sequence into the corresponding numbers according to the one-to-one correspondence between letters and numbers (A->0, T->1, C->2, G->3). One-hot coding, in fact, means that there are N state registers used to encode N states. Each state has an independent register bit, and only one of these register bits is valid. In other words, there can only be one state. This method ignores the relationship between adjacent sequences.

As shown in Table 3, one-hot encoding scheme showed poor effect in every metric. Adjacent sequences are separated in this process and coding these sequences by one-hot into the EBLSTM may not be a good idea. The other three methods have a similar effect by careful observation, and SN of letters to numbers and 3-mer is equal. But in other metrics, 3-mer is undoubtedly the best one. Similarly, as shown in Table 4, in the process of enhancer classification, the difference among different encoding schemes will be more obvious. It can be seen that 3-mer performs better than the others for each item; thus, we think 3-mer is a more suitable encoding method for this experiment.

**TABLE 3:** Result of comparison of using different encoding schemes in layer 1 (enhancers identification) under 10 trials.

Encoding scheme	ACC	AUC	SN	SP	MCC
Letters to numbers	0.753	0.824	0.755	0.750	0.500
One-hot	0.565	0.611	0.494	0.642	0.132
2-Mer	0.758	0.827	0.735	0.762	0.505
3-Mer	0.772	0.835	0.755	0.795	0.534
					

**TABLE 4:** Result of comparison of using different encoding schemes in layer 2 (enhancers classification) under 10 trials.

Encoding scheme	ACC	AUC	SN	SP	MCC
Letters to numbers	0.640	0.650	0.784	0.512	0.302
One-hot	0.526	0.522	0.438	0.412	0.116
2-Mer	0.645	0.662	0.786	0.498	0.304
3-Mer	0.658	0.688	0.812	0.536	0.324
					

### Comparison of Different Architectures

In this experiment, we tried eight different internal structures, including simple RNN, bidirectional RNN, simple LSTM, and bidirectional LSTM, and then, on the basis of the four networks doubled, respectively, which means that another four structures are two layers of RNNs, bidirectional RNNs, simple LSTMs, and bidirectional LSTMs. After this step, a model that has the best performance would be chosen that with higher metrics than other seven models. Then the dropout layer is added to produce the final architecture.

Tables 5, 6 show the different architecture results in layers 1 and 2, respectively. The results are shown from the results in Table 5. Except for SN, the bidirectional LSTM has better effect based on the four other evaluation metrics. The reasons may be that bidirectional LSTM is more complex than the other three architectures and more features can be captured by it. In fact, we also do the other four experiments, as mentioned in the previous paragraph. But increasing the number of layers in this architecture also raises the processing time longer. The efficiency will be reduced. Therefore, the results of these four experiments were added to the table. A similar situation occurs in Table 6, where bidirectional LSTM is also the better choice in many metrics, except SP. Together, these results provide important insights into the idea that bidirectional LSTM is the best fit for the experiment.

**TABLE 5:** Result of comparison of using different architectures in layer 1 (enhancers identification) under 10 trials.

Architecture type	ACC	AUC	SN	SP	MCC
Simple RNN	0.721	0.791	0.732	0.760	0.488
Bidirectional RNN	0.745	0.801	0.767	0.751	0.492
Simple LSTM	0.742	0.812	0.802	0.746	0.512
Bidirectional LSTM	0.772	0.835	0.755	0.795	0.534
					

**TABLE 6:** Result of comparison of using different architectures in layer 2 (enhancers classification) under 10 trials.

Architecture type	ACC	AUC	SN	SP	MCC
Simple RNN	0.617	0.634	0.801	0.591	0.249
Bidirectional RNN	0.628	0.617	0.792	0.612	0.276
Simple LSTM	0.634	0.626	0.770	0.578	0.302
Bidirectional LSTM	0.658	0.688	0.812	0.536	0.324

### Comparison of Different Ensemble Models

As mentioned in Section “Ensemble Model,” during the experiment, we tested three ensemble strategies. Each method has advantages and disadvantages. To explore which kind of strategy is more suitable for enhancers DNA sequences characteristics identification, median, voting, and averaging are tested. Set of indicators across the different methods are assessed. In Table 7, the voting and averaging methods are significantly better than the median method, and their performance of the two is very similar, but AUC and MCC in the averaging method are higher than those in the voting method, which shows that the predictive effect and stability of the average method are more advantageous than those of the voting method. In addition, in Table 8, the averaging method is still the best of these three ensemble methods. Combining these two tables to draw a conclusion, the indicators for the averaging method are better than the other two methods. The averaging method is the best one, and finally in our model, this method is applied to achieve ensemble learning.

**TABLE 7:** Result of comparison of using different ensemble models in layer 1 (enhancers identification) under 10 trials.

Ensemble method	ACC	AUC	SN	SP	MCC
Median	0.728	0.788	0.774	0.726	0.498
Voting	0.765	0.762	0.792	0.738	0.517
Averaging	0.772	0.835	0.755	0.795	0.534
					

**TABLE 8:** Result of comparison of using different ensemble models in layer 2 (enhancers classification) under 10 trials.

Ensemble method	ACC	AUC	SN	SP	MCC
Median	0.622	0.664	0.740	0.572	0.310
Voting	0.638	0.644	0.794	0.562	0.311
Averaging	0.658	0.688	0.812	0.536	0.324
					

### Comparison With Existing State-of-the-Art Methods

There are several excellent methods for the prediction of enhancers, and the well-known methods are iEnhancer-2L, EnhancerPred, iEnhancer-EL, and iEnhancer-ECNN. Tables 9, 10 show the results of the comparison with existing state-of-the-art methods in layers 1 and 2.

**TABLE 9:** Result of comparison with existing state-of-the-art methods in layer 1 (enhancers identification).

Method	ACC	AUC	SN	SP	MCC	Source
iEnhancer-2L	0.730	0.806	0.710	0.750	0.460	Liu B. et al., 2016
EnhancerPred	0.740	0.801	0.735	0.745	0.480	Jia and He, 2016
iEnhancer-EL	0.748	0.817	0.710	0.785	0.496	Liu B. et al., 2016; Liu G. et al., 2018
iEnhancer-ECNN	0.769	0.832	0.785	0.752	0.537	Nguyen et al., 2019
iEnhancer-EBLSTM	0.772	0.835	0.755	0.795	0.534	This study
						

**TABLE 10:** Result of comparison with existing state-of-the-art methods in layer 2 (enhancers classification).

Method	ACC	AUC	SN	SP	MCC	Source
iEnhancer-2L	0.605	0.668	0.470	0.740	0.218	Liu G. et al., 2016
EnhancerPred	0.550	0.579	0.450	0.650	0.102	Jia and He, 2016
iEnhancer-EL	0.610	0.680	0.540	0.680	0.222	Liu G. et al., 2018
iEnhancer-ECNN	0.678	0.748	0.791	0.564	0.368	Nguyen et al., 2019
iEnhancer-EBLSTM	0.658	0.688	0.812	0.536	0.324	This study

As Table 9 shows, compared with the previous three experimental methods, all the results of the metrics are significantly improved, especially in AUC and MCC. Moreover, compared with iEnhancer-ECNN in 2019, in this study, the results for ACC, AUC, and SP are slightly higher, but the results for SN and MCC are slightly lower. As seen in Table 10, iEnhancer-EBLSTM remains a reliable method that has better performance than iEnhancer-2L, iEnhancer-EL, and EnhancerPred, especially for SN and MCC; this method has been greatly improved. From the experimental results, we can see that both IEnhancer-EBLSTM and IEnhancer-ECNN are significantly better than the previous methods. I think the reason lies in the fact that the deep learning model itself has certain advantages, which can capture features more accurately and learn more efficiently. The model obtained can have more accurate parameters, so as to obtain higher results. However, compared with iEnhancer-ECNN, the data for AUC in our experiment are lower than the result of them, but the data for SN are higher. Overall, these results indicate that iEnhancer-EBLSTM performs best in enhancer identification and classification.

## Discussion

In this paper, we proposed the prediction model called iEnhancer-EBLSTM to identify enhancers and their strengths. In addition, this model uses the principle of 3-mer to encode the DNA sequence and EBLSTM to get the predictive result. The biggest advantage of this method is that it only uses DNA sequence information and does not rely on other features such as chromosome status, gene expression data, and histone modification. This greatly facilitates researchers to use it. iEnhancer-BLSTM could be used not only for identifying enhancers but also for distinguishing strong enhancers from weak enhancers. In the first layer, the predictor can identify whether the DNA sequence is enhancer or not, and the ACC is 0.772. In the second layer, the predictor can classify that the enhancer is strong or weak, and the ACC is 0.658. A lot of work still needs to be done on the second layer. There is little difference between strong and weak enhancers. More and more information of DNA sequences, physical and chemical needs to be mined. The characteristic information can be recorded more completely, and the various models can be built based on this information in more detail.

## Data Availability Statement

The original contributions presented in the study are included in the article/supplementary material, further inquiries can be directed to the corresponding author/s.

## Author Contributions

YZ conceived and designed the project. KN and XL conducted the experiments and analyzed the data. KN and YZ wrote the manuscript. TZ and YZ revised the manuscript. All authors read and approved the final manuscript.

## Conflict of Interest

The authors declare that the research was conducted in the absence of any commercial or financial relationships that could be construed as a potential conflict of interest.
